# DNA methylation molecular subtypes for prognosis prediction in lung adenocarcinoma

**DOI:** 10.1186/s12890-022-01924-0

**Published:** 2022-04-07

**Authors:** Duoduo Xu, Cheng Li, Youjing Zhang, Jizhou Zhang

**Affiliations:** 1 Wenzhou Hospital of Traditional Chinese Medicine Affiliated to Zhejiang Chinese Medicine University, No. 9 Jiaowei Road, Lucheng District, Wenzhou City, Zhejiang Province China; 2 Department of Otolaryngology Head and Neck Surgery, The Central Hospital of Wuhan, Tongji Medical College Huazhong University of Science and Technology, Wuhan, Hubei China; 3 School of Public Health, Tongji Medical College, Huazhong University of Science and Technology, Wuhan, China

**Keywords:** LUAD, DNA methylation, TCGA, Prognosis

## Abstract

**Aims:**

Lung cancer is one of the main results in tumor-related mortality. Methylation differences reflect critical biological features of the etiology of LUAD and affect prognosis.

**Methods:**

In the present study, we constructed a prediction prognostic model integrating various DNA methylation used high-throughput omics data for improved prognostic evaluation.

**Results:**

Overall 21,120 methylation sites were identified in the training dataset. Overall, 237 promoter genes were identified by genomic annotation of 205 CpG loci. We used Akakike Information Criteria (AIC) to obtain the validity of data fitting, but to prevent overfitting. After AIC clustering, specific methylation sites of cg19224164 and cg22085335 were left. Prognostic analysis showed a significant difference among the two groups (*P* = 0.017). In particular, the hypermethylated group had a poor prognosis, suggesting that these methylation sites may be a marker of prognosis.

**Conclusion:**

The model might help in the identification of unknown biomarkers in predicting patient prognosis in LUAD.

**Supplementary Information:**

The online version contains supplementary material available at 10.1186/s12890-022-01924-0.

## Background

Lung cancer is one of the main results in tumor-related mortality, and in China, it ranks first and the second highest cause of cancer morbidity among men and women, respectively [1, 2]. Some of the associated factors causing increased lung cancer mortality include increased tobacco use, aging, and atmospheric pollution. In 2018, lung cancer was predicted to have caused 1.8 million deaths [3–5]. The general subtype of lung cancer is lung adenocarcinoma (LUAD) and due to its late recognized, the five-year survival rate is reported to be 15% [6]. LUAD is sensitive to chemotherapy, however, the rapid increase in drug resistance and chemo-resistance result in the death of most patients [7, 8]. For advanced-stage LUAD, molecularly targeted therapies are reported to increase the patients' survival rates. however, many more patients failed to have a useful targetable mutation. LUAD usually initiates from abnormal hyperplasia of the bronchial mucosa, continued by malignant infiltration and growth. Epigenetic alteration is closely associate to tumorigenesis, growth, and metastasis, but DNA methylation raised forward constantly in the complex course of tumorigenesis and acts as key part in regulating gene function in tumor cells. Methylation differences reflect critical biological features of the etiology of LUAD and affect prognosis [9]. In this way, it is necessary for recognizing the more useful novel specific epigenetic targets for developing powerful prognostic evaluation, survival, and timely launch of therapeutic drugs and treatment ways of LUAD.

DNA methylation characterized to the response catalyzed through DNA methyltransferase (DNMT), whereby a methyl of S-adenosylmethionine (SAM) is transferred to the cytosine form five-methylcytosine (5-mC). It mainly arises in the C-phosphate-G site (CpGs) and over two thirds of mammalian CpGs are methylated [10]. While, un-methylated CpGs tend to aggregate in the seed sequence of structural gene promoters as well as transcription start sites (TSS), forming CpG islands [11]. CpG islands exist in the 5′ regulatory area of most genes, especially in the promoter region. The high level of 5-methylcytosine in the gene promoter area can lead to gene silencing in tumor cells, and then suppresses gene expression, leading to cell dysfunction. Chromosomal instability, particularly 17p loss, offer proof for the accumulation of mutations and propose that cancerous regions are helpful for the selection and expansion of these precancerous lesions in LUAD [12]. Currently, methylation of some promoter sequences, involving *EGFR*, *KRAS*, *TP53*, *ECT2*, *S100A16*, and *AGTR1* has been related to the incident and development of LUAD [13–15]. But, the value of these gene methylations in clinical not well examined in LUAD patients. Besides, there is currently no systematic assessment of predictions of overall survival (OS) or characteristics involve in DNA methylation in LUAD. In the present study, we built a prediction prognostic signature integrating DNA methylation used high-throughput omics data for improved prognostic evaluation.

## Materials and methods

### Data gathering

We downloaded RNA-Seq standardized FPKM data and clinical comparison data from 486 cases in the TCGA-LUAD (www. portal.gdc.cancer.gov/). Methylation data from Illumina Infinium was got from the UCSC Cancer Browse (www. genome.ucsc.edu/) and human methylation was performed on 27 and 450 bead chip arrays in cases from 150 and 503 patients, respectively. The level of each methylation site is expressed as a β value, ranging from1 (fully methylated) to 0 (unmethylated). CpG loci for which data were lacking over 70% of the subjects were rule out. The cross-reactive genome CpG sites in "Illumina Infinium Human Methylated 450-bead Array Discovery of Cross Reaction Probe and Polymorphic CpG" were not included. The k-nearest neighbor (KNN) estimator is applied for the remaining sites where input data is unable available. Combat algorithms in the R SVA package eliminate the batching effect. Unsteady genomic sites, involving CpG and individual nucleotide polymorphisms on sex chromosomes, were eliminated. Because gene expression regulated promoter DNA methylation, we specifically studied the promoter region CpGs (from transcription start site 2 kb upstream to 0.5 kb downstream). In addition, we choose cases with available gene expression profiles. Overall, 458 subjects and 21,120 methyl sites were contained. Samples are divided into two groups: a testing set (27 Beadchip) and a training set (450 Beadchip).

### Identification of classification features

CpG loci that significantly affected survival were applied as classification features. Univariate Cox regression model was established based on the methylation level, TNM, age, sex, stage and survival information. Significant CpGs got from the univariate Cox regression model were included in the multivariate Cox regression model. Then CpG loci were selected as characteristic CpG loci. The risk score was acquired based on the formula: e^sum (CpG's expression×coefficient)^. The way of prediction accuracy of the prognostic signatures used by ROC curve.

### Identification of molecular subtypes

ConsensusClusterPlus (R package) was used for consensus clustering, and the LUAD subgroups were identified according to the CpG sites that varied the most. The algorithm first subsamples some items and features from the data matrix, and each subsample is divided into group K by kmeans. The "consensus" clustering is defined by calculating the stability of the clustering results from using a specific clustering way to a random data subset. The area under the curve with no obvious change was taken as the classification count. To bring more thorough classification categories for LUAD, more categories are tended to be used. Use color gradients to act for consensus values from zero (white) to one (dark blue); arrange the matrices so that items being classified the alike cluster are next to each other.

### Prognosis and Function analyses

Kaplan–Meier diagrams are applied to demonstrate OS in the LUAD subgroup defined by DNA methylation profiles. The significance of the differences among clusters evaluate by log-rank test. The relationship between clinical, biological characteristics and DNA methylation clusters analyzed by chi-square test. KEGG and Genetic Ontology (GO) (Molecular Functions (MF), Biological Processes (BP), and Cellular Components (CC)) for the analysis of biological functions and annotation genes using the R ggplot2 package to display the graph. All tests were bilateral, and *P* < 0.05 was considered statistically significant.

## Results

### Built the prognostic methylation sites signatures

**Fig. 1:**
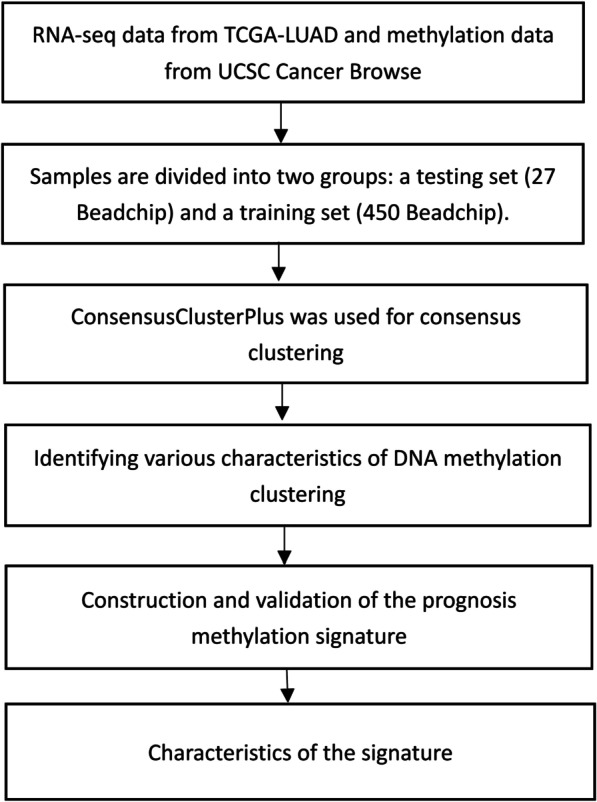
A flow chart of the study

A flow chart of this study was shown in Fig. 1. In the training dataset, overall 21,120 methylation sites were identified. The univariate Cox regression identified 1103 CpG sites as prospective DNA methylation biomarkers for OS. Cox regression analysis of the 864 methylation sites with tumor stage, sex, TMN, and age as covariates identified 205 independent prognosis-related CpG sites. The clinicopathological properties of the samples are shown in Table 1. Middle-aged at diagnosis was 60.5 years, and the median age of final exposure of the study subjects was 9.3 years.

**Table 1:** TCGA lung adenocarcinoma patient characteristics

Clinical characteristics	Total (486)	%
Age at diagnosis (y)	60.5 (33–88)	
Futime (y)	9.3 (0–18.7)	
Gender		
Female	265	54.5
Male	221	45.5
Stage		
I	262	53.9
II	112	23.1
III	79	16.3
IV	25	5.1
NA	8	1.6
Grade		
NA	486	100
T-classification		
T1	163	33.5
T2	260	53.5
T3	41	8.4
T4	19	3.9
TX	3	0.6
M-classification		
M0	333	68.5
M1	24	4.9
MX	125	25.7
NA	4	0.8
N-classification		
N0	312	64.2
N1	90	18.5
N2	70	14.4
N3	2	0.4
NX	11	2.3
NA	1	0.2
Status		
Alive	162	33.3
Death	324	66.7

Data express as mean (min–max)

### Identify DNA methylation subgroups

**Fig. 2:**
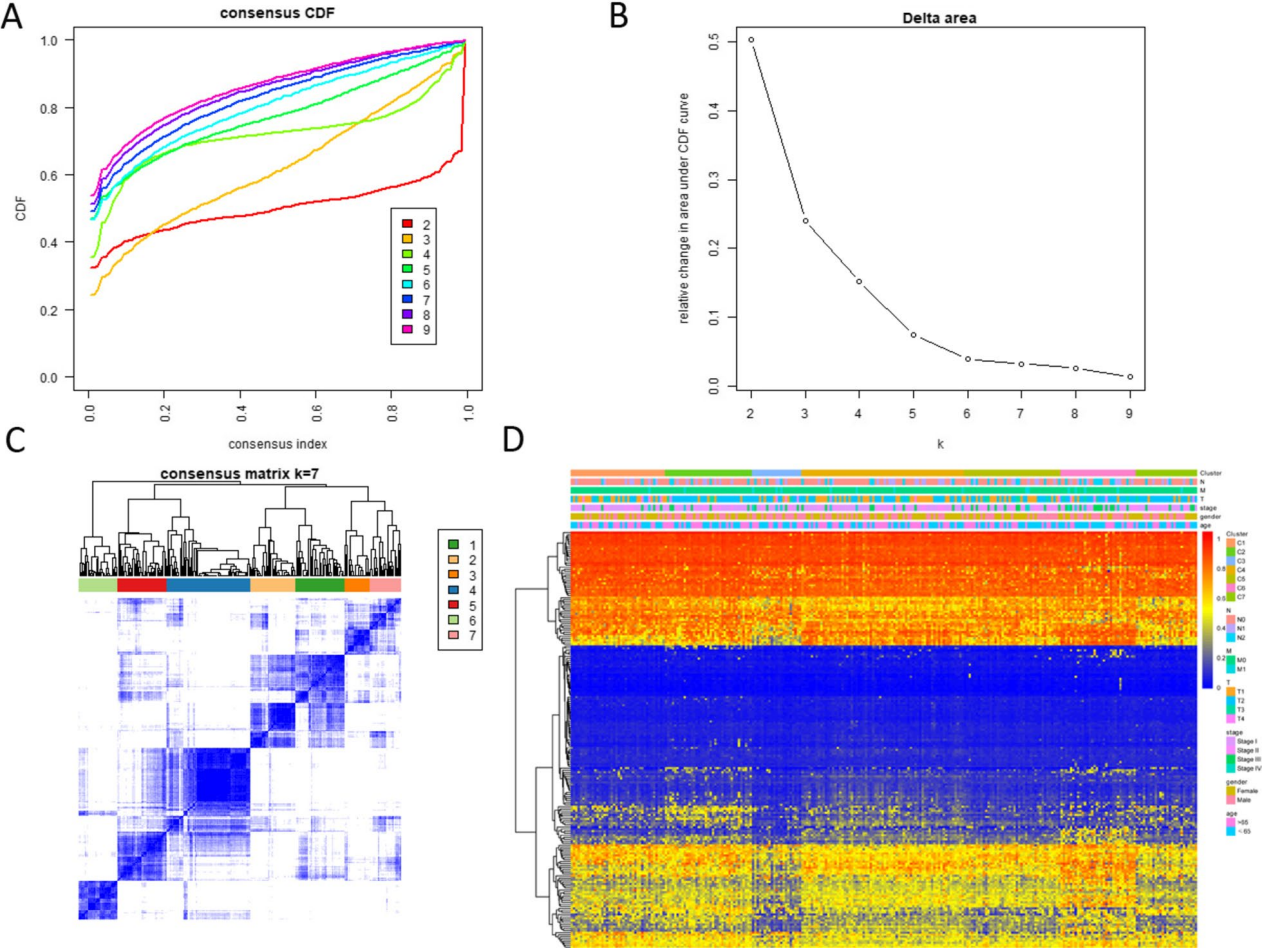
Criteria for selecting a number of categories and consensus matrix for DNA methylation classification with the corresponding heat map. **A** The consensus among clusters for each category number k. **B** Delta area curves for consensus clustering indicating the relative change in area under the CDF curve for each category number k compared to k − 1. The horizontal axis represents the category number k and the vertical axis represents the relative change in area under the CDF curve. **C** Color-coded heatmap corresponding to the consensus matrix for k = 7 obtained by applying consensus clustering. Color gradients represent consensus values from 0 to 1; white corresponds to 0 and dark blue to 1. **D** A heatmap corresponding to the dendrogram in **C** was generated using the heatmap function with DNA methylation classification, TNM stage, clinicopathological stage, and histological type as the annotations

**Fig. 3:**
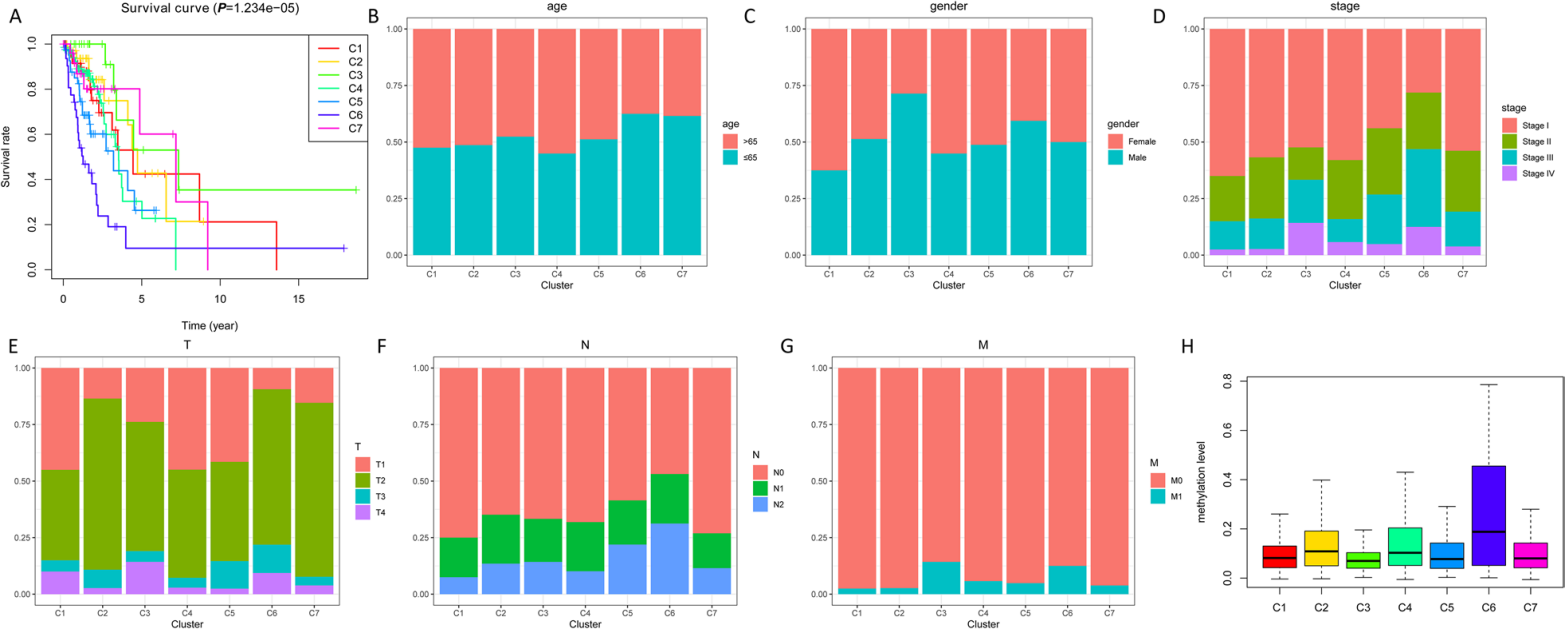
Comparison of prognosis and clinical factors between the DNA methylation clusters. **A** Survival curves for each DNA methylation subtype in the training set. Age (**B**), gender (**C**), Stage score (**D**), topography score (**E**), lymphocyte infiltration (**F**), and metastasis (**G**) distributions for each DNA methylation subtype in the training set. **H** Box plot of CpG methylation levels of the 7 Clusters

A consensus cluster of the 205 possible prognostic methylation sites was applied to identified molecular subgroups of DNA methylation. Under the region of the Cumulative Distribution Function (CDF) curve as well as a consensus, matrix to decide the numbers of clusters. The CDF curve began to stabilize afterward cluster 6 (Fig. 2A, B). To increase the prognostic worth of the LUAD subgroups, we choose even more cluster number when possible. The consensus matrix indicates the consensus for k = seven illustrates a well-determined seven-block pattern Fig. 2C. The corresponding heat map marked with TNM, gender, stage, age and DNA methylation subgroup in the tree diagram of Fig. 2C is shown in Fig. 2D. OS analysis illustrated that there was a statistically significant difference in prognosis among the seven groups (*P* < 0.05). Cluster 6 had the worst prognosis, but cluster three and seven had the best prognosis (Fig. 3A). Then, we analyzed the clustering proportions of the seven clusters based on TNM, stage, gender and age Fig. 3B–G. The relationship between characteristics and specific clusters is: Clusters 1, 4 and 5 with lower T-level; Group 5 and group 6 were advanced group; Clusters 1 and 7 of lower N rank; Class M and above Group 3 and 6; Group 4 was older, group 1 had more women, and group 3 had more men. These results suggest that each clinical factor is related to different intra-cluster rates.

### Identifying various feature of DNA methylation clustering

**Fig. 4:**
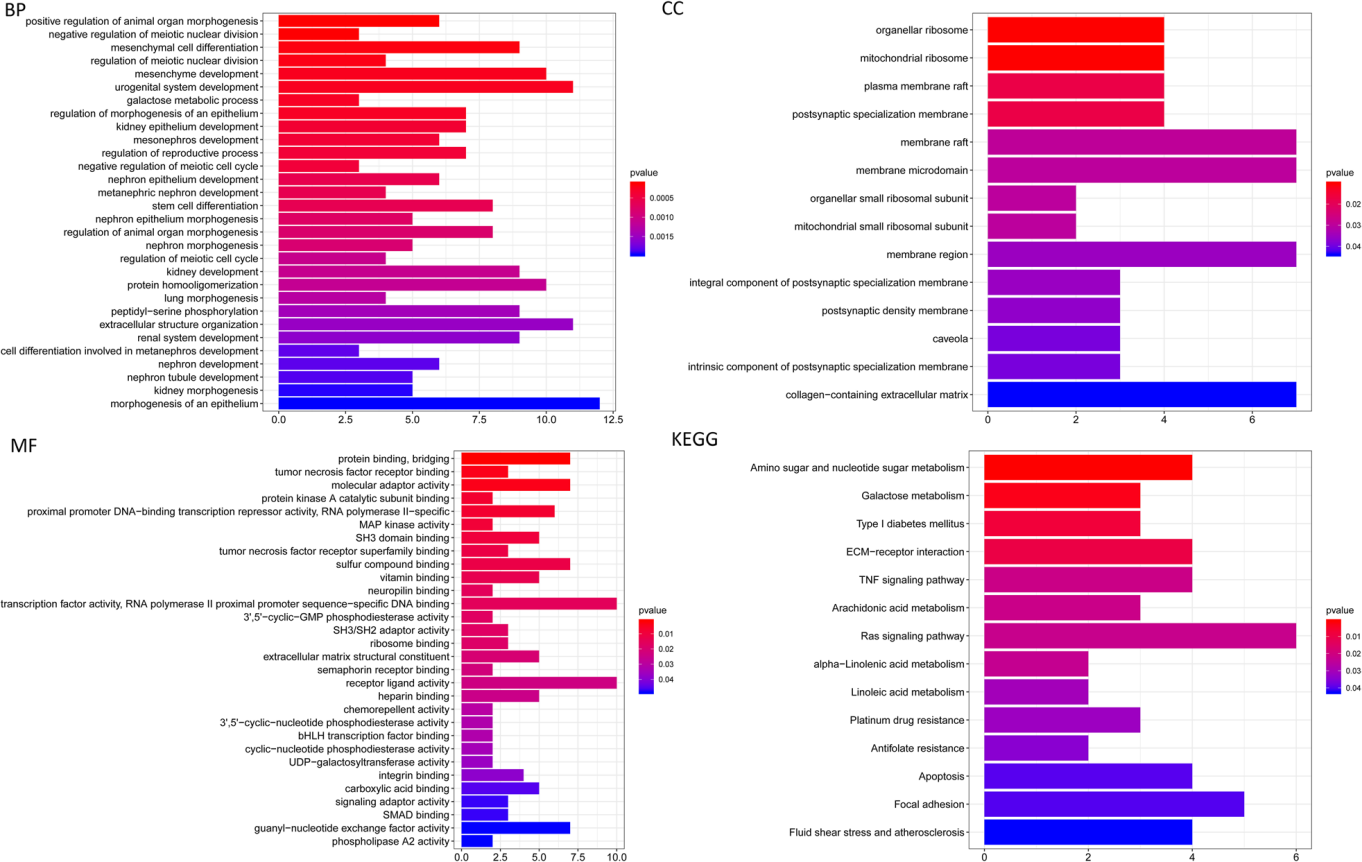
The function of the identified CpG sites corresponding promotor genes using gene ontology (GO) enrichment and KEGG pathway analysis

**Fig. 5:**
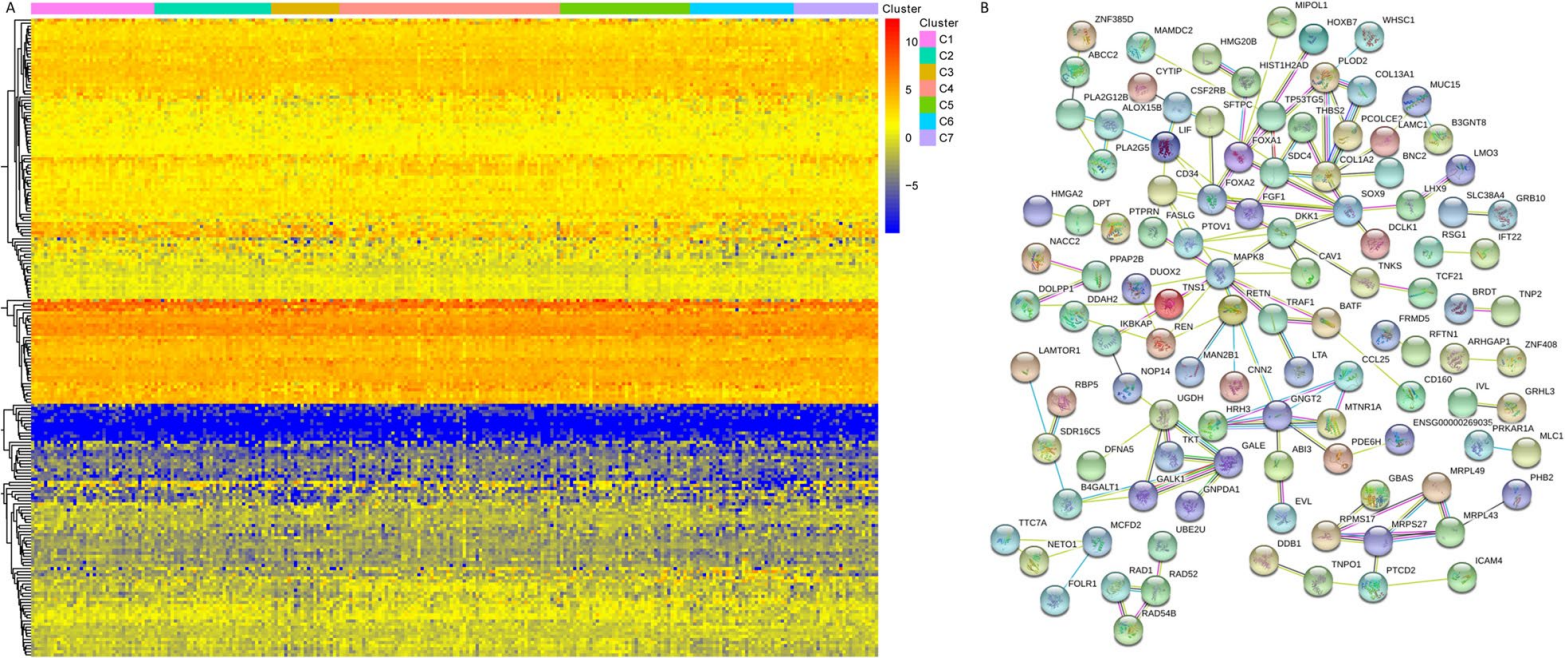
Gene annotations of the 205 methylated sites. **A** Cluster analysis heat map for annotated genes associated with the 205 CpG site. **B** The PPI network for annotated genes associated with the 205 CpG site

A total of 237 promoter genes were identified by genomic annotation of 205 CpG loci. Then we used the software package "Clusterprofiler" R to perform functional enrichment analysis on the 237 genes. BP associated pathways were mainly enriched in the regulation of animal organ morphogenesis, meiotic nuclear division, cell differentiation, and some metabolic process. MF associated pathways were significantly enriched in protein/factor binding, bridging, kinase/receptor-ligand activity. CC associated pathways were mainly enriched in the ribosome, postsynaptic specialization membrane, and other membrane regions. KEGG pathway was mainly enriched in metabolism pathways, Platinum drug resistance, Antifolate resistance, Apoptosis, ECM-receptor interaction, TNF and Ras signaling pathway (Fig. 4). We then analyzed the expression of the methylated genes identified in the subgroup, and the heatmap of gene expression is shown in Fig. 5A. Gene expression models varied among the subgroups an indication that the level of DNA methylation reflected the expression of the genes. A protein–protein interaction network was constructed and four hub genes (*CCL25*, *PRMS17*, *NETO1*, and *RAD1*) were identified using the MCODE of Cytoscape as shown in Fig. 5B. We then explored for cluster-specific methylation sites by using the methylation sites as cluster characteristics. To |log_2_FC|> 1 joint *P* value < 0.05 as selection criteria, will be one of the clusters as a single cluster, six other clustering as different, the difference between 7 clustering analysis. Cluster 6 had the highest specific sites, most of which were hypermethylated, and the level of methylation was the highest among all clusters (Fig. 3H).

### Constructing and verifying the signature

**Fig. 6:**
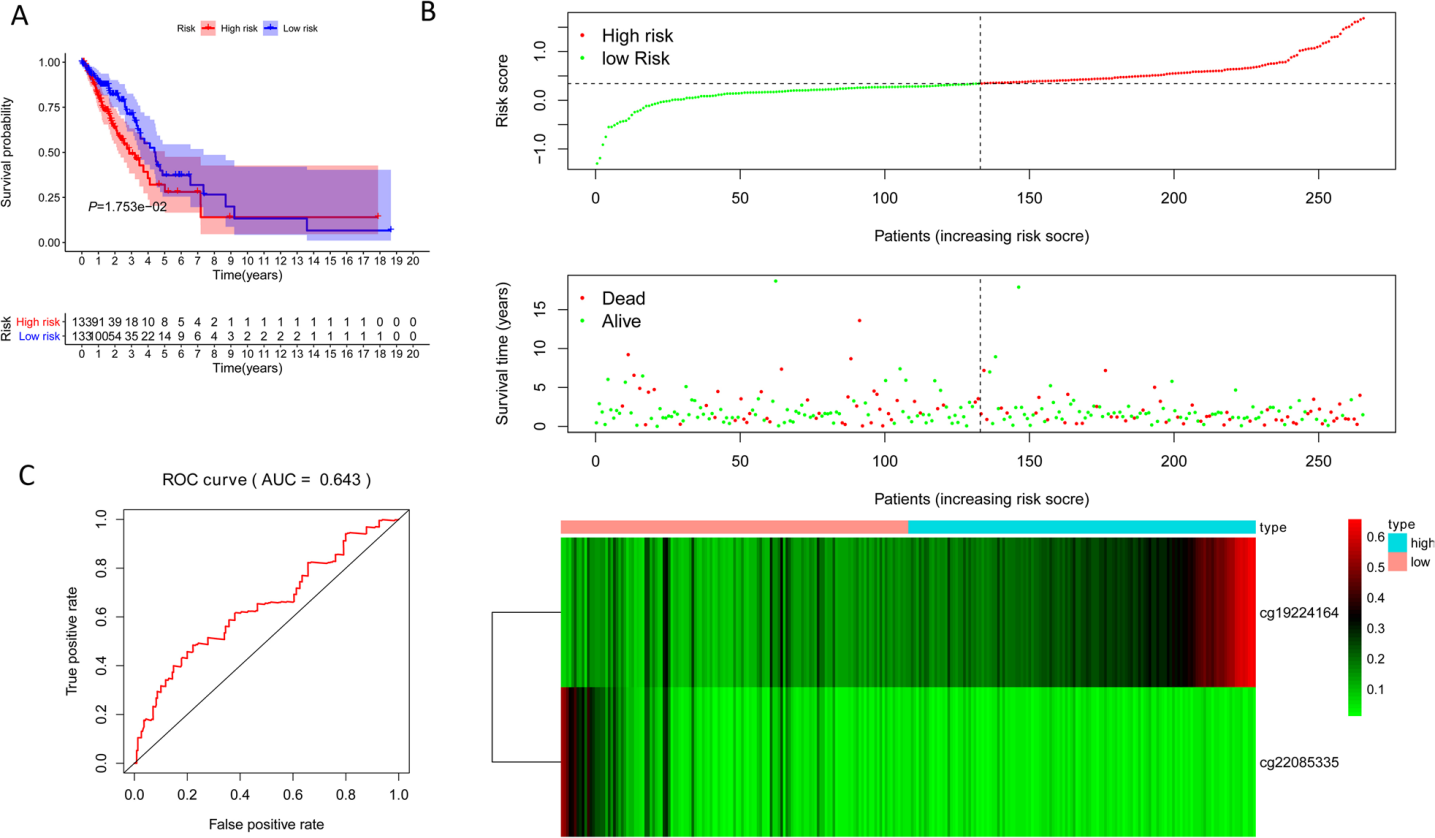
Construction of the prognosis prediction model for the training set LUAD patients. **A** Analysis of prognostic differences after classification in the training set. **B** The horizontal axis represents the samples, and the vertical axis represents risk scores (top), overall survival (middle), and methylation site (bottom). **C** ROC curves of prognostic predictors in LUAD patients

**Fig. 7:**
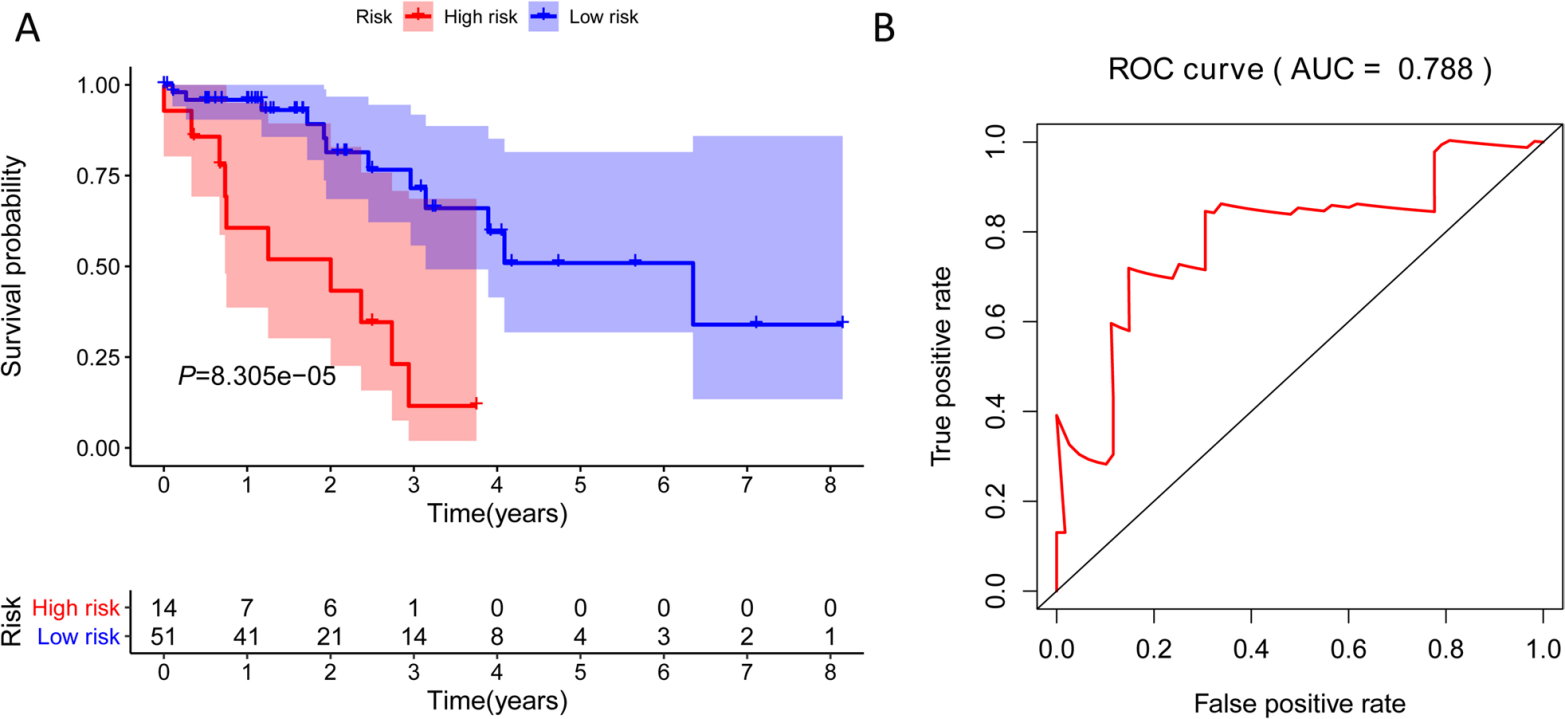
The prognosis prediction model in testing set. **A** Overall survival analysis. **B** ROC curves results

Cluster 6 was chosen as the seed cluster as it had the most specific methylation sites. Cluster 6 has 7 specific methylation sites, among which cg22085335 is hypomethylation site and the others are hypermethylation sites. We used Akakike Information Criteria (AIC) to obtain the validity of data fitting, but to prevent overfitting to the greatest extent. After AIC clustering, specific methylation sites of cg19224164 and cg22085335 were left. Therefore, our model formula is as follows: risk score = 2.71*cg19224164 + 2.75*cg22085335. Using the median risk score as the threshold, the samples were divided into two (high-risk vs. low-risk) groups. Prognostic analysis illustrated a significant difference among the two groups (*P* = 0.017), as shown in Fig. 6A. The samples were then sorted according to the risk score to determine whether methylation levels changed systematically as the risk score changed, as shown in Fig. 6B. In particular, the hypermethylated group had a poor prognosis, suggesting that these specific methylation sites may be a marker of prognosis. Area under the curve (AUC) is 0.643, indicating normal operation of the model Fig. 6C. The methylation levels at specific sites increased significantly as the risk score increased. At last, a test data set to predict patient prognostic outcomes. There was also a significant difference in prognosis between the two groups (Fig. 7A, P = 8.305E−05). The AUC of the test sample is 0.788, suggesting that the model runs well Fig. 7B. Since EGFR mutations are major driver gene mutations, we also found a positive correlated with the methylation status of cg19224164 and cg22085335 Additional file 1: Fig. S1. In addition, the impacts of risk scores on patient OS in different clinical subtypes were explored, and the results indicated that Female, Stage III–IV, and T1–2 subtypes were significantly correlated with the survival of a patient Additional file 2: Fig. S2. Further, the differences in somatic mutations between the low and high-risk groups was also explored and *TTN* was the most common mutated gene Additional file 3: Fig. S3. These findings are consistent with the results of the training data set, which proves the stability and accuracy of our model prediction.

## Discussion

Lung cancer is the most critical lead to cancer-related mortality worldwide, resulting in over one million deaths each year. Adenocarcinoma is the most prevailing histological subtype of non-small cell lung cancer. Smoking is the leading result to lung adenocarcinoma. But, as the number of smokers has decreased in many countries, the incidence of LUAD among non-smokers has raised. Even if the 5-year survival rate of LUAD has get better in recent years owing to advances in surgical treatment, radiation therapy, and chemotherapy, it is still dissatisfactory. In order to enhance the administration of LUAD, molecular definition-based methods usually do not demand huge tissue samples, which can increase patient volume and reduce unnecessary surgical step. DNA methylation plays an important role in epigenetic function by reducing the activity of DNA fragments and inhibiting gene transcription.

The use of DNA methylation markers can do us a better prognosis and predict therapy response, thereby extending patient survival. DNA methylation changes are an early event in tumorigenesis and are crucial in the regulation of gene expression in cancers. Therefore, in the early diagnosis of LUAD, epigenetic changes can be detected either independent or in combination with other traditional biomarkers [16]. One study obtained eight probes corresponding to the characteristics of eight genes (*AGTRL1*, *CTSE*, *EFNA2*, *ALDH1A3*, *BDKRB1*, *NFAM1*, *TMEM129*, and *SEMA4a*) to predict survival in patients with early LUAD, but this study only included Asian and Caucasian populations [17]. In another study, 6 differentially methylated genes (*JDP2*, *PLG*, *SERPINA5*, *SEMG2*, *RFX5*, and *POLR3B*) were identified to predict the prognosis of LUAD patients, but the study restricts in stage I patients [18]. Abnormal methylated genes may serve as non-invasive biomarkers for diagnosis at early, treatment selection, response assessment and possible applications of novel therapies. We identified 205 independents prognostic CpG loci and 237 corresponding promoter genes. We also built a PPI network and determined four central genes (*CCL25*, *PRMS17*, *NETO1*, and *RAD1*). C–C motif chemokine ligand 25 (*CCL25*), belongs to the subfamily of small cytokine CC genes and the product of this gene binds to chemokine receptor *CCR9*. *CCR9-CCL25* axis is reported to play a critical role in breast cancer (BC) cell survival and low chemotherapeutic effect of cisplatin primarily via *PI3K/Akt* dependent fashion [19]. Progesterone receptor modulators (PRMs) constitute an interesting new hormone drug for BC treatment, and anti-proliferative effects of various PRMs have been reported [20]. *NETO1* regulates *NMDAR* and kainic acid receptor (KAR) to control synaptic transmission by acting as a helper protein for two types of ionic glutamate receptors in a synaptic-specific manner [21]. The Rad9-Hus1-Rad1 protein complex is thought to respond to DNA destruction and play an indispensable role in the cell cycle [22]. *RAD9* inhibition can potentiate the cytotoxic reaction of chemotherapy on BC cells [23]. Besides, mouse *RAD1* deletion is reported to enhance sensitivity for skin tumor development probably by maintaining genomic integrity [24]. Currently, there is no comprehensive study on the role of *CCL25*, *PRMS17*, *NETO1*, and *RAD1* in LUAD and this study may provide key information to in-depth studies.

KEGG pathway enrichment analysis indicated that they were mainly enriched in metabolism pathways, Platinum drug resistance, Antifolate resistance, Apoptosis, ECM-receptor interaction, TNF/Ras signaling pathway. Stage II–IIIA LUAD patients generally accept platinum-based ACT after surgical resection, while just 4–15% survival advantage after adjuvant chemotherapy (ACT) has been observed [25]. Van et al. constructed a 37-gene signature for identifying patients with longer and shorter survival after receiving platinum-based ACT and then determined them to non-responders and responders, respectively [26]. Thus, we hypothesis that the 237 promotor genes act as a key role in Platinum drug resistance and need further research. Although methylation may essential in LUAD, specific methylation sequences in the promoter region affecting gene expression remain unclear. Besides, in a larger group of LUAD patients, the statistical and clinical significance of these gene methylation associated to prognosis needs to be demonstrated. In this study, we tried to develop a classification model integrating many DNA methylation biomarkers to evaluate the prognosis. This model can promote the determination of novel biomarkers, molecular subtype classification, and precise medical targets of diseases in LUAD. Meanwhile, the model can also help with prognosis prediction, diagnosis, and strategies of patients with difference epigenetic subtypes of LUAD.

The signatures might give DNA methylation alteration and offer potentially useful targets for cancer treatment and prediction therapy response. But, our signatures have to prove in further independent studies as well as predictive DNA methylation functional by experiments. This study has limitations. First, the results have not yet been validated in clinical samples. Second, these results do not offer precise clinical data as a result of the relatively small sample size of patients used. Finally, due to the limited data, we could not discuss the role of cg19224164 and cg22085335 and the role of tobacco and alcohol habit information in LUD. Although our study hopes to explore the possibility of establishing predictive models, it is still in its infancy and needs to be improved. Meanwhile, cg19224164 and cg22085335 not only may be a useful biomarker but also a potential therapeutic target in LUAD.

## Conclusion

In summary, prognostic specific methylation sites were identified by TCGA database and other bioinformatics methods, and a prognostic prediction model was constructed for LUAD patients. The model can help identify novel biomarkers, predict prognosis, clinically diagnose and manage patients with different distinct subtypes of LUAD.

## Supplementary Information

**Additional file 1. Fig. S1**: The correction among EGFR mutations with the methylation status of CG19224164 and CG22085335.

**Additional file 2. Fig. S2**: The impacts of risk scores on patient OS in different clinical subtypes.

**Additional file 3. Fig. S3**. The differences in somatic mutations between the low and high-risk groups.

**Additional file 4. Table S1**: The univariate Cox regression shown 1103 CpG sites were identified as potential DNA methylation biomarkers for OS in LUAD patients. **Table S2**: The multivariate Cox regression analysis of the 864 methylation sites with T, N, M, stage, gender, and age as covariates recognized 205 independent prognosis-related CpG sites. **Table S3**: The genomic annotation of the above 205 CpG sites was used to identify overall 237 corresponding promoter genes. **Table S4**: The functional enrichment analysis of these 237 genes. **Table S5**: The differences expression among the 7 clusters.

## Data Availability

All data were downloaded from public databases, containing The Cancer Genome Atlas (TCGA, https://tcga-data.nci.nih.gov/tcga/) and the Gene Expression Omnibus (GEO, http://www.ncbi.nlm.nih.gov/geo/).

## Abbreviations

LUAD: Lung adenocarcinoma
TCGA: The Cancer Genome Atlas database
