# Supplementary figures and images for: DNA methylation molecular subtypes for prognosis prediction in lung adenocarcinoma
Source: BMC Pulm Med. 2022 Apr 7;22:133. doi: 10.1186/s12890-022-01924-0 (PMC8991665; doi:10.1186/s12890-022-01924-0)

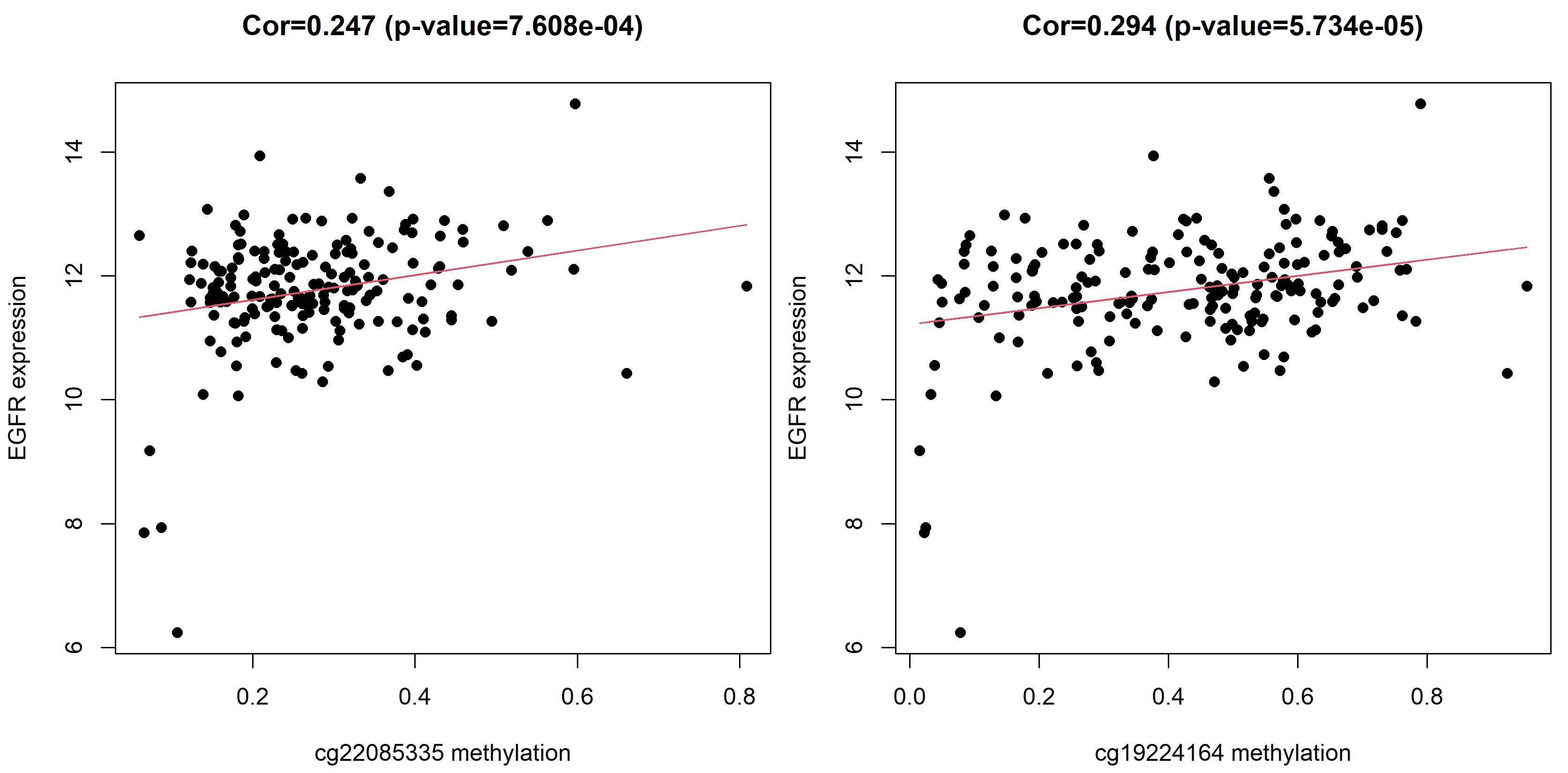

Supplement: Supplementary file 1 — Additional file 1. Fig. S1: The correction among EGFR mutations with the methylation status of CG19224164 and CG22085335. [file 12890_2022_1924_MOESM1_ESM.jpg]

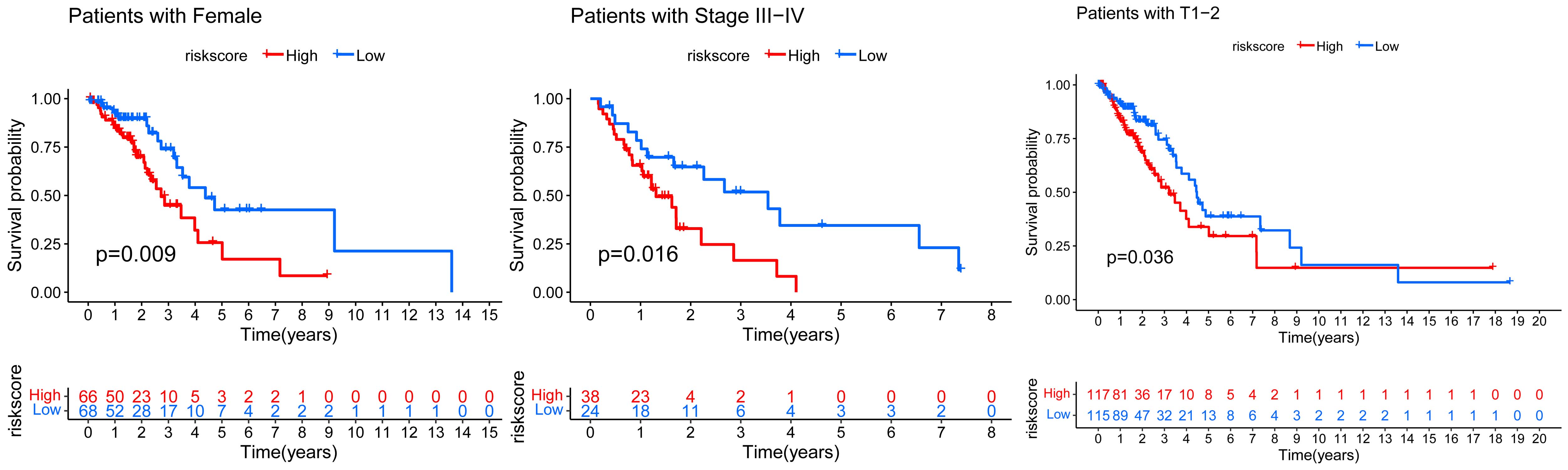

Supplement: Supplementary file 2 — Additional file 2. Fig. S2: The impacts of risk scores on patient OS in different clinical subtypes. [file 12890_2022_1924_MOESM2_ESM.jpg]

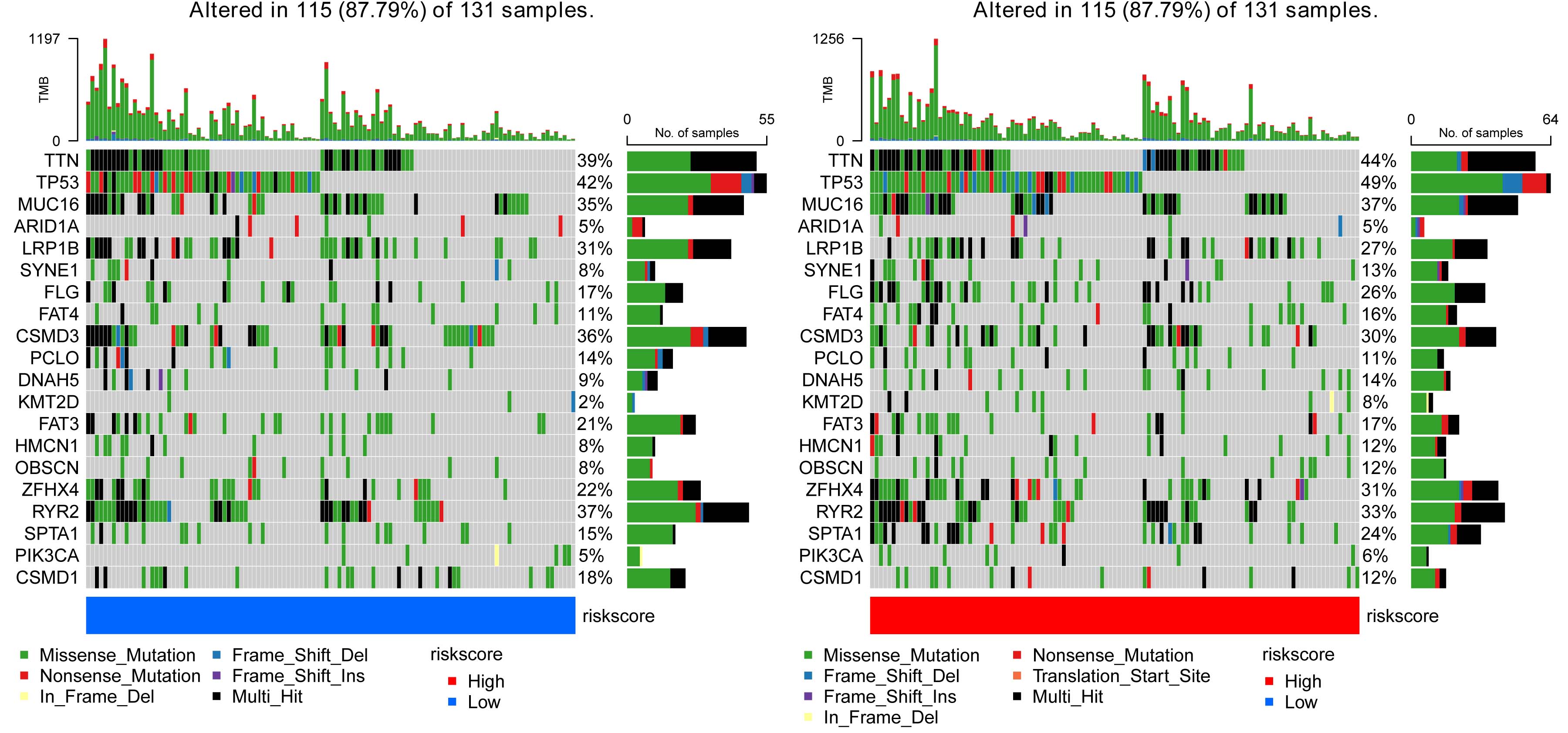

Supplement: Supplementary file 3 — Additional file 3. Fig. S3. The differences in somatic mutations between the low and high-risk groups. [file 12890_2022_1924_MOESM3_ESM.jpg]
